# Supplementary material for: Synchronization of speed, sound and iridescent color in a hummingbird aerial courtship dive
Source: Nat Commun. 2018 Dec 18;9:5260. doi: 10.1038/s41467-018-07562-7 (PMC6299134; doi:10.1038/s41467-018-07562-7)
Supplement: Supplementary file 1 — Supplementary Information [file 41467_2018_7562_MOESM1_ESM.pdf]

Supplementary Information for:

Synchronization of speed, sound and iridescent color in a hummingbird aerial courtship dive

Benedict G. Hogan and Mary Caswell Stoddard

Contact: Mary Caswell Stoddard

Email: mstoddard@princeton.edu

This PDF file includes:

Supplementary Methods and Notes 1-3

Supplementary Figures 1-10

Supplementary Tables 1-3

Supplementary References

Other supplementary materials for this manuscript include the following:

Supplementary Data 1

Supplementary Methods

Sound synchronization procedure

Because several results of the paper hinge on the sycnronization between recorded sound and captured footage, we undertook the following procedure to check and amend the synchronization of the captured video and extracted audio recordings. The GoPro camera was placed on a table in a suitably quiet indoor area. Four two-minute-long videos were taken using the same settings used in the field. Every 10 seconds, an object was dropped onto the table in view of the camera. The footage was then linearized and sound was extracted, as in the main paper. The same procedure used to track birds (using dltv5 [1]) was used to find the frames in the footage that corresponded to the impact of the object on the table. Separately, the timing of the noise generated by this impact was measured by hand-assigning points on a sonogram of the audio from the footage, using Matlab's 'sonogram' function. These times were then correlated. We found that the audio was consistently 0.053s (~6.36 video frames) behind the video. Correcting for this by removing the first 0.053s of audio in all recordings left us with a mean synchronization error of 4.6*10^-5s and a standard deviation of 0.031s (n=50).

In addition, sounds emanating from the male bird take some time to reach the camera. This delay changed slightly over the course of the dive as the distance from the camera to the male changed. For each dive we estimated this delay. For a dive 9m away from the camera the delay was estimated to be 0.026s with the male at the nadir of the dive (with sound of speed = 343.14m s^{-1}). The estimated mean maximum delay was 0.054s, where the male was at the top of the dive (n=48, SD = 0.011). Because the differences between delays are fairly small, and vary over and between dives, and since the key measured times all occur near the nadir of the dive, we took into account only the delay estimated at the nadir, and thus removed the first 0.026s of audio in all recordings. Omitting or including this correction did not qualitatively affect the results. In the main paper, the influence of the Doppler effect was calculated with Supplementary Equation 1.

Supplemental Equation 1

$$Doppler\ shift = \frac{Speed\ of\ sound}{Speed\ of\ sound - relative\ velocity}$$

Where the speed of sound was assumed to be 343.14 m s^{-1} (speed of sound in dry air at 20° C), and relative velocity is the velocity of the male in relation to the female.

Perspective correction/spatial calibration procedure

During data collection in the field, the camera was located on the ground and was tilted upwards to capture the greatest extent of dives possible. The birds were recorded diving orthogonal to the camera, but dives can reach heights of around 30m. This means that our linearized footage does not represent the actual position of the bird, but the position of the bird subject to perspective. To account for this, and to move from image-space (in pixels) to real-world space (in m), videos were taken of the side of a building, which presented landmarks that allowed us to know real distances within the images. The building was 22m wide and 11m tall, with rectangular landmarks which were 4.4m wide and 2.2m tall. We took videos of the building at

the same distance used in the main paper. Each video contained a meter stick vertically oriented against the building, and in each video the camera was rotated in pitch. This allowed us to find frames in each video that most closely matched the viewing geometry of the footage taken in the field, by reference to the length of the meter stick (in pixels) and the distance (in pixels) from the base of the meter stick to the bottom of the image. The best matching images were linearized using the same camera parameters as the main paper and are taken as 'calibration images.'

Thirty-six landmarks in the calibration images were digitized, using the same methods as the main paper. We then used Matlab's 'fitgeotrans' command to find the best-fitting projective transformation from the pixel values to the known real-world meter positions of the points. For each dive in the main paper, the digitized bird x-y positions were transformed using the resultant transformation matrix. See Supplemental Figure 1 for an illustration of these calibration images, and of the calibration image before and after this transformation.

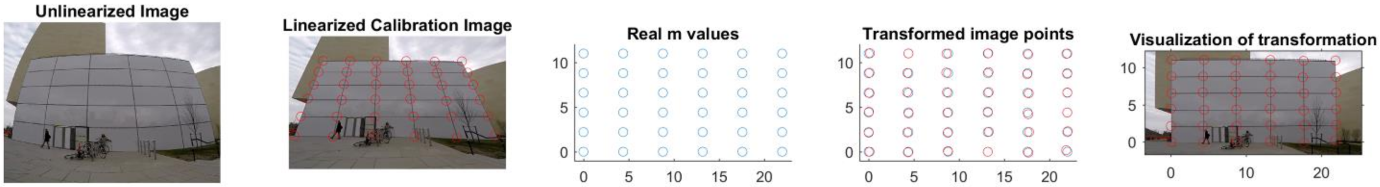

Supplementary Figure 1 – Illustration of the perspective correction/spatial calibration procedure. Left: Un-linearized footage of the building. Middle left: The same frame linearized, with landmarks digitized. Middle: Scatterplot of the real-world positions of those landmarks. Middle right: transformation of the digitized landmarks to the real-world positions. Right: Visualization of the transformation generated by transformation of the calibration image and digitized landmarks.

Female view

Using estimated female position and assumptions about male orientation (see main paper, and below), we estimated the geometry of the scene from the perspective of the female. The angle at which the female saw the male (*i.e.*, his relative orientation) was calculated by adding the four-quadrant inverse tangent of the female and male positions (remapped to 0-360°) to the instantaneous orientation of the male.

We also estimated the visual angle that the male bird takes up in the female's field of view, calculated using Supplementary Equation 2.

Supplementary Equation 2

$$Visual\ angle = 2Arctan\left(\frac{Size}{2Distance}\right)$$

Where distance is the distance between the male and female, and size is the size of the male as seen at his orientation relative to the female (see above), with the size of a male assumed to be 8.5cm (the estimated length of the male excluding the beak [2], though note that because the main paper only makes use of relative extent of visual angle the size used does not influence the results). While this approach simplifies the male to a line and therefore ignores his depth

and width, only his length is of importance to understand at what point in the dive he encompasses the female’s maximal visual angle. This is because while at the bottom of the dive the male travels horizontally above the female and does not face her.

Supplementary Note 1

Female position

Current information about the position of females relative to dives indicates that she is under the nadir of the dive [3,4]. We tested the robustness of our findings to variation in female position. All procedures were as in the main paper, except that the position of the female relative to the nadir of the dive was modified. We present data for three female positions, plotted alongside measures using the female position used in the main paper (0.5m below the nadir), to facilitate comparison. The female positions tested were 1.5m below the nadir of the dive (representing a 1m shift in female position 'downward' relative to the position used in the main paper), 0.5m below but 1m preceding the nadir of the dive (so the male passes the female before he reaches the nadir of the dive), and 0.5m below and 1m following the nadir of the dive.

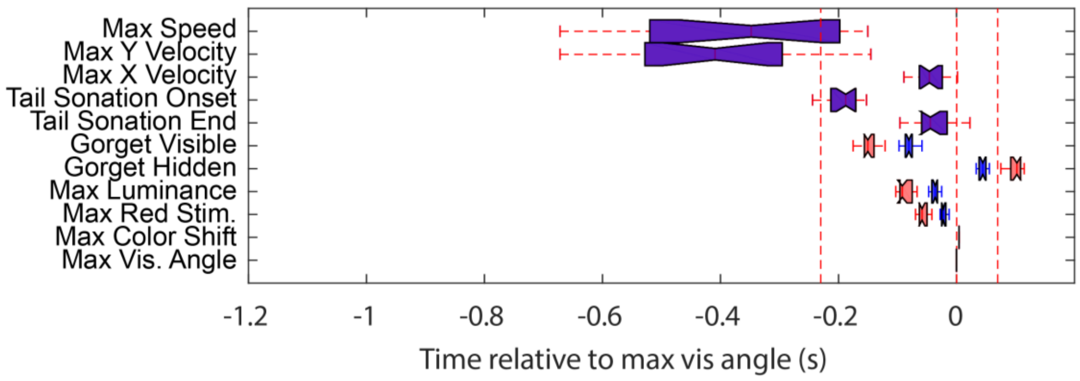

Supplementary Figure 2. Box plot of key features of the dive: mean time of maximal estimated speed, mean time of maximal Y (vertical) and X (horizontal) velocity, mean times of tail-generated sonation onset and cessation, mean times of gorget visibility, mean time of maximal estimated luminance of the gorget, mean time of maximal estimated female LWS stimulation, mean time of maximal color shift, and the mean time of maximal female visual angle taken up by the male. Plotted in blue are values for female position used in the main paper (female position x=0m, y=-0.5m relative to the nadir of the dive); plotted in red are values when the female was moved vertically away from the nadir of the dive by 1m (female position x=0m, y=-1.5m), color appears purple where measures overlap.

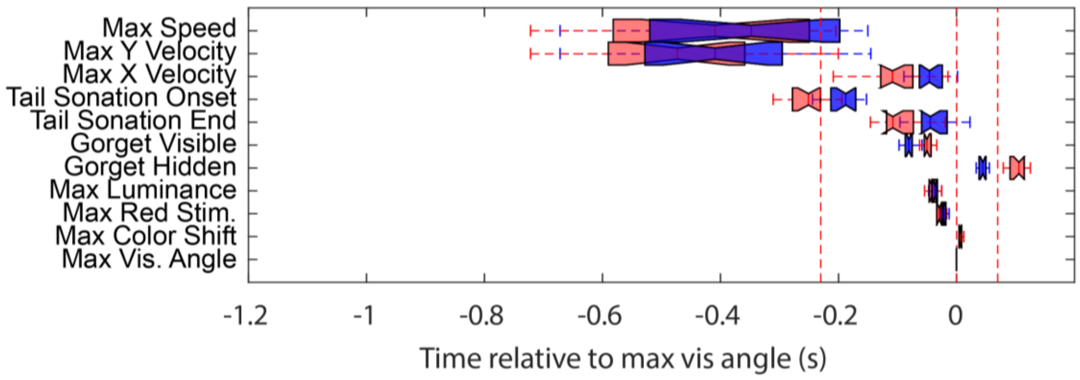

Supplementary Figure 3. Box plot of key features of the dive: mean time of maximal estimated speed, mean time of maximal Y (vertical) and X (horizontal) velocity, mean times of tail-generated sonation onset and cessation, mean times of gorget visibility, mean time of maximal estimated luminance of the gorget, mean time of maximal estimated female LWS stimulation, mean time of maximal color shift, and the mean time of maximal female visual angle taken up by the male. Plotted in blue are values for female position used in the main paper (female position x=0m, y=-0.5m relative to the nadir of the dive); plotted in red are values when the female was moved horizontally away from the nadir by 1m, such that the male passes her after the nadir of the dive (female position x=-1m, y=-0.5m). Purple color indicates overlap between red and blue box plots.

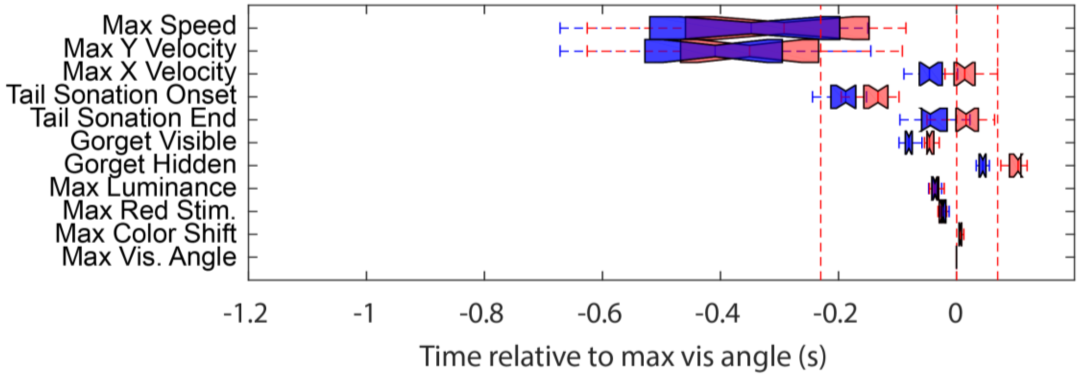

Supplementary Figure 4. Box plot of key features of the dive: mean time of maximal estimated speed, mean time of maximal Y (vertical) and X (horizontal) velocity, mean times of tail-generated sonation onset and cessation, mean times of gorget visibility, mean time of maximal estimated luminance of the gorget, mean time of maximal estimated female LWS stimulation, mean time of maximal color shift, and the mean time of maximal female visual angle taken up by the male. Plotted in blue are values for female position used in the main paper (female position x=0m, y=-0.5m relative to the nadir of the dive); plotted in red are values when the female was moved horizontally away from the nadir by 1m, such that the male passes her before the nadir of the dive (female position x=1m, y=-0.5m). Purple color indicates overlap between red and blue box plots.

The temporal organization of key components of the dive is somewhat sensitive to female position; however, even these relatively large displacements of the female do not disrupt the general synchronization of the time of maximal horizontal velocity, the timing of the tail-generated sonations and the visibility of the gorget. A dive that passes less closely to the female (see Supplemental Figure 2) has no effect on relative timing of maximal speed, velocity or sonations, but does influence the visual components. The gorget is visible for a greater duration for a dive that passes further from the female, and this change is reflected in the timing of peak LWS stimulation and peak luminance, although not in the time of maximal color shift (because color change is always most rapid around the time the female is normal to the male, see main text Fig. 2). However, while the gorget is visible for longer – it must take up a smaller absolute degree of the visual field of the female. This could indicate an interesting trade-off, wherein males may face a conflict between displaying their gorgets for longer, or getting as close to the female as possible. Additionally, the perceived amplitude of the male's sonations will decrease exponentially as the distance between his dive and the female increases.

When the female is displaced such that the male passes her after he has passed the nadir of the dive, the visual components of the dive are largely unchanged (relative to the female position used in the main paper.) However, the time at which the male reaches maximal speed and (both horizontal and vertical) velocity, and the time at which the male emits tail-generated sonations – relative to the time at which he takes up the greatest degree of female visual field – are affected (see Supplemental Figure 3). In this case, we see that these events (as expected) now fall further before the time of maximal visual angle. That is, they are less well synchronized with the time at which the gorget is visible.

When the female is instead displaced 1m in the other direction, such that the male passes over her before he reaches the nadir of the dive, the inverse is true (see Supplemental Figure 4). The timing of maximal speed, (horizontal and vertical) velocity, and tail-generated sonations are shifted closer toward the time of maximal visual angle. It is interesting to note that this movement in female position apparently increases the degree of synchronization between the acoustic and visual components of the dive. In this study, we rely on the literature to generate an assumed female position. However, it seems likely that the ability of the male to accurately locate the female, and to use her as a reference point during the dive, will vary. Since that variability is here shown to influence the temporal organization of the dive, it may well be an important factor in the signaling behavior. Equally, this finding could lead to testable hypotheses about female position relative to male dives. For instance, we could generate the hypothesis that the male will attempt to perfectly synchronize the tail-generated sonations to the time during which his gorget is visible. We would then predict that the male should position the dive such that he passes the female ~>1m before he passes the nadir of the dive.

Supplementary Note 2

Male orientation

For the purposes of the current study, the posture of the male was assumed to be fixed, with his beak's orientation coincident with that of his body and his body is facing his direction of travel at all points. This assumption does not hold during the ascending part of the dive, when he may be facing other directions – but the descending part of the dive was the focus of the study presented. To our knowledge, changes in the posture of broad-tail hummingbird males, apart from the spreading of tail feathers in order to produce tail-generated sonations, during the descending or horizontal parts of the dive are not documented. However, there remains the possibility that the male orients his head or body to increase the visibility of the gorget to the female during the dive, for instance by holding his beak upward during these parts of the dive. Small changes in the orientation of the head relative to the body are known in Anna's hummingbird (although in the opposite direction, where the beak is downward relative to the body for a short period of the dive [5]). In order to examine the possible effects of changes in posture that change the period for which the gorget is visible to the female, we undertook a similar test to the one reported above. In this case, we can simulate postural changes by artificially increasing the angles at which the gorget can be seen. We found in the main paper that the gorget is only visible between -80º and 60º (where 0º is normal to the long axis of the male). Here we re-run the analysis while extending these angles to -90º to 70º, -100º to 80º, and -110º to 90º, increasing the range of angles from which the gorget is visible by 20º, 40º, and 60º, respectively.

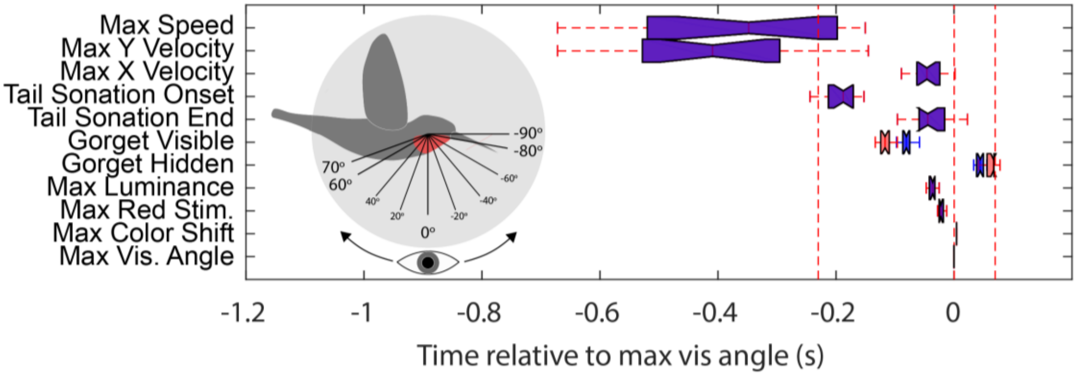

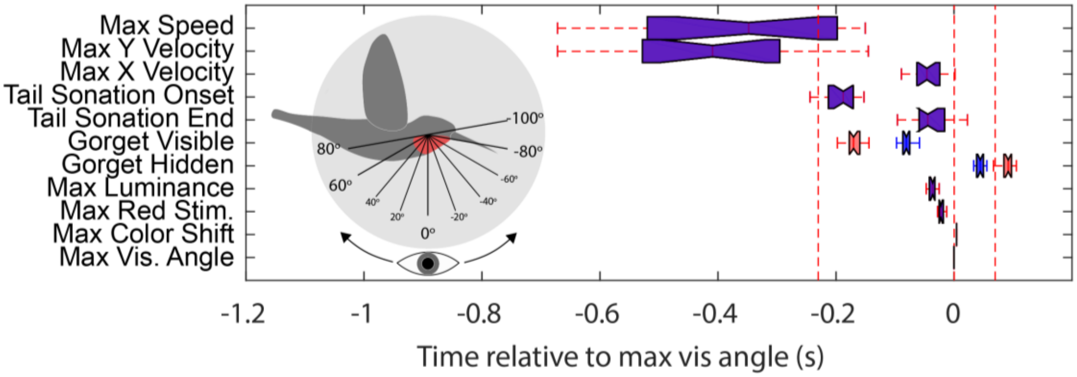

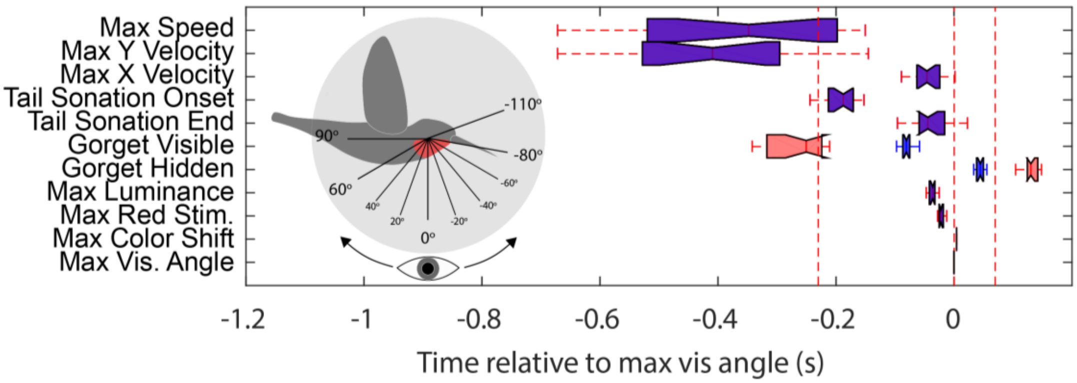

Supplementary Figure 5 - Box plots of key features of the dive: mean time of maximal estimated speed, mean time of maximal Y (vertical) and X (horizontal) velocity, mean times of tail-generated sonation onset and cessation, mean times of gorget visibility, mean time of maximal estimated luminance of the gorget, mean time of maximal estimated female LWS stimulation, mean time of maximal color shift, and the mean time of maximal female visual angle taken up by the male. Plotted in blue are the values when the angular extent of the visibility of the gorget is as in the main paper (-80º to 60º). Plotted in red are values when the angular extent is modified. Top: -90º to 70º. Middle: -100º to 80º. Bottom: -110º to 90º.

We found that changes in male posture could influence the duration for which his gorget is visible to the female during the dive. However, even a fairly drastic increase of 60º (30º in each direction) does not disrupt the overall scheme of the results. As before, the timing of the visibility of the gorget is temporally aligned with the nadir of the dive, and therefore also with the tail-generated sonations and time of maximal horizontal velocity.

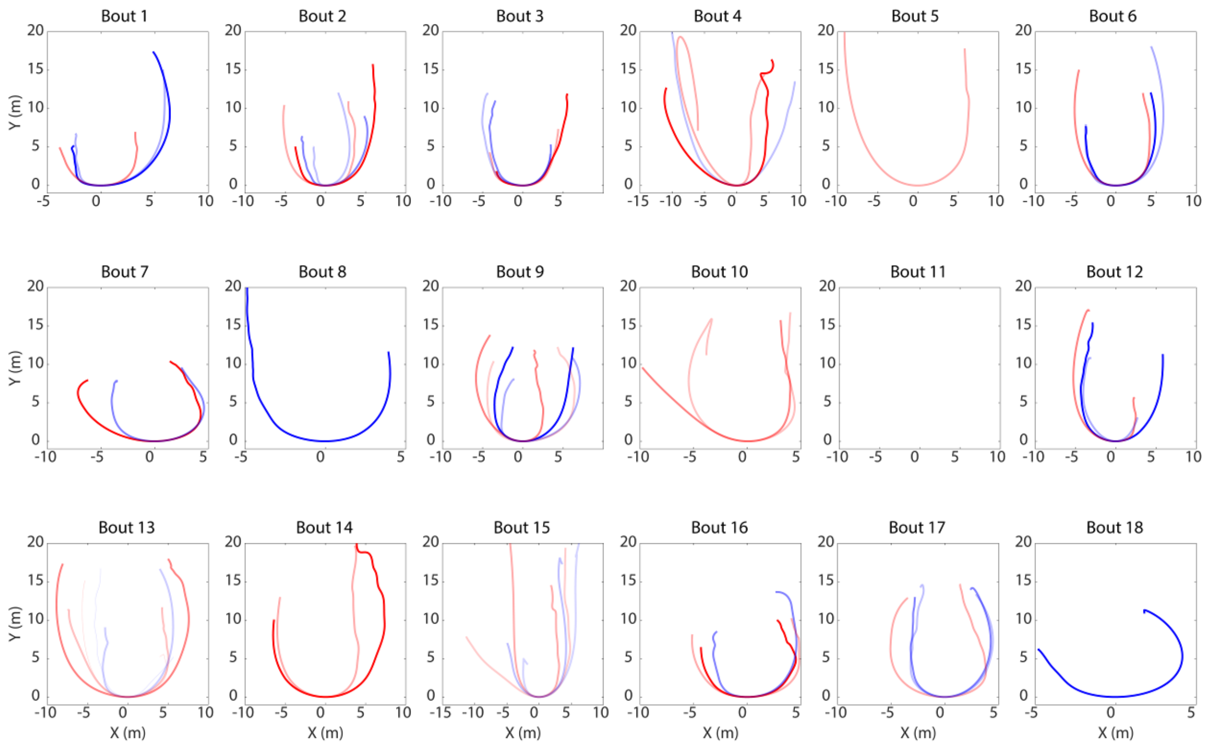

Supplementary Figure 6. Tracked dives separated by video to indicate symmetry of dives within bouts of diving. Red lines indicate right to left movement, blue lines indicate left to right movement. Opacity decreases with successive dives.

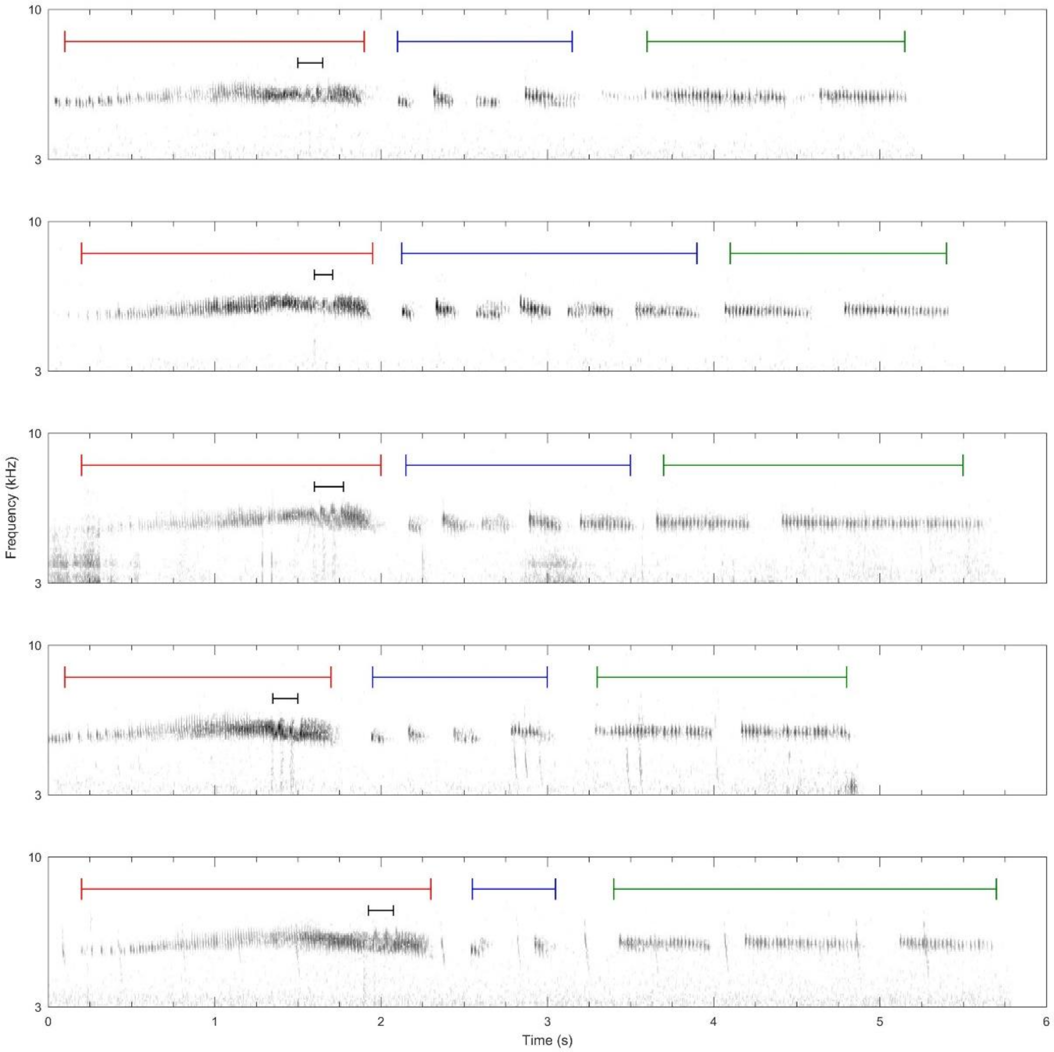

Supplementary Figure 7. Example sonograms from recorded dives with sections of dive sonations annotated. Red: wing-generated main dive sound, blue: wing-generated short sections, green: wing-generated long sections, black: tail-generated sonations. Note that female vocalizations can also be seen in the lower two sonograms

Supplementary Table 1 – Mean and standard deviation of relative cone stimulation predicted from the gorgets of ten male broad-tailed hummingbird museum specimens measured using UV/VIS photography, as a function of viewing angle (where the normal of the male bird is 0º, and negative values indicate rotation of the beak toward the observer). Luminance: double cone stimulation and standard deviation. UVS: ultraviolet-sensitive cone stimulation and standard deviation. SWS: shortwave-sensitive cone stimulation and standard deviation. MSW: mediumwave-sensitive cone stimulation and standard deviation. LWS: longwave-sensitive cone stimulation and standard deviation.

Pitch	N	Mean Luminance	STD Luminance	Mean UVS	STD UVS	Mean SWS	STD SWS	Mean MWS	STD MWS	Mean LWS	STD LWS
-80	10	0.1291	0.0339	0.1867	0.0271	0.1654	0.0157	0.1748	0.0327	0.4731	0.0373
-60	10	0.1524	0.0658	0.1867	0.0186	0.1708	0.0163	0.1484	0.0264	0.494	0.0243
-40	10	0.1802	0.0422	0.1986	0.0118	0.1829	0.0139	0.1472	0.0205	0.4713	0.0204
-20	10	0.1186	0.0273	0.1898	0.0105	0.1827	0.0115	0.1812	0.0225	0.4463	0.0217
0	10	0.0683	0.015	0.195	0.0073	0.1894	0.0064	0.2365	0.015	0.3791	0.0186
20	10	0.042	0.0054	0.2133	0.0081	0.1988	0.0029	0.2711	0.0051	0.3168	0.0105
40	10	0.0334	0.0045	0.2264	0.0101	0.1993	0.0044	0.2767	0.0054	0.2976	0.0072
60	10	0.0299	0.0053	0.2393	0.0156	0.1955	0.0119	0.2767	0.0051	0.2885	0.0072

Supplementary Table 2 – Just noticeable differences between the mean relative cone stimulations of museum specimen gorgets at varied viewing angles. Values calculated using Tetracolorspace [6],with a weber fraction of 0.05 [7], and relative cone density from peafowl (*Pavo cristatus*; 0.46:0.85:1:0.96) [8].

Pitch	60	40	20	0	-20	-40	-60	-80
60	-	1.0025	2.4374	6.8220	12.3597	15.7629	16.4344	13.8582
40	1.0025	-	1.5224	6.1005	11.7770	15.2745	15.9152	13.3122
20	2.4374	1.5224	-	4.6757	10.4944	14.1212	14.6866	12.0227
0	6.822	6.1005	4.6757	-	6.0276	9.9234	10.2583	7.4691
-20	12.3597	11.7770	10.4944	6.0276	-	4.0685	4.2503	2.1440
-40	15.7629	15.2745	14.1212	9.9234	4.0685	-	1.7372	3.9325
-60	16.4344	15.9152	14.6866	10.258	4.2503	1.7372	-	3.2716
-80	13.8582	13.3122	12.0227	7.4691	2.1440	3.9325	3.2716	-

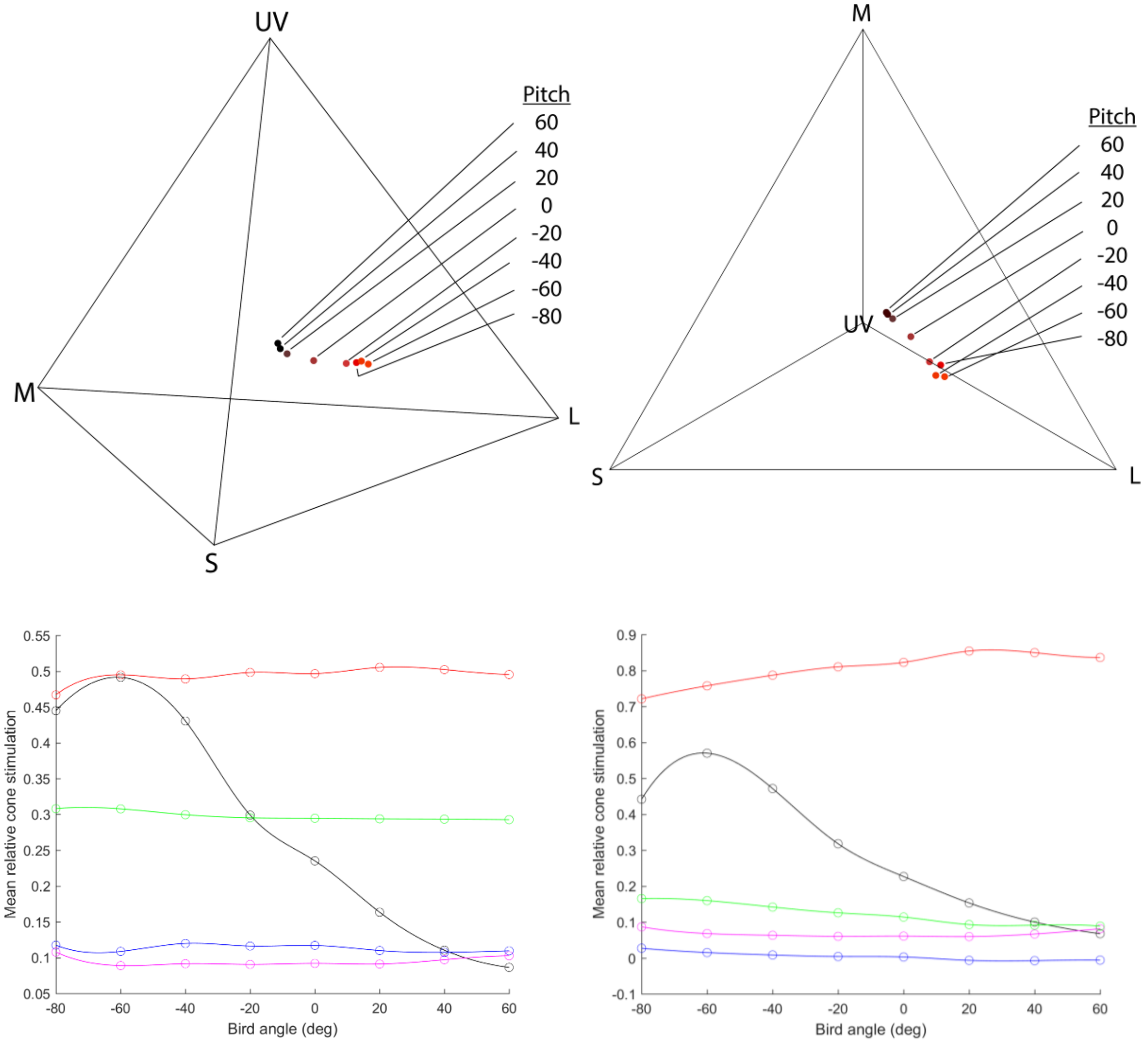

Supplementary Figure 8. Top panel: Mean broad-tailed hummingbird gorget color as predicted by a hummingbird vision model (see main text) plotted in tetrahedral color space. Left and right panels represent different views of the same data. Points within the tetrahedron indicate mean relative stimulation from gorgets at different viewing angles. UV: ultraviolet-sensitive cone stimulation. S: shortwave-sensitive cone stimulation. M: mediumwave-sensitive cone stimulation. L: longwave-sensitive cone stimulation. Bottom panel: Mean relative cone stimulation of European robin (*Erithacus rubecula*) breast (left), and black-winged bishop (*Euplectes hordeaceus*) gorget (right), as predicted by a hummingbird vision model as a function of male orienation. Note that negative x values indicate that the beak is rotated toward the observer and vice versa. Circles indicate measured values, lines show smoothing spline interpolation. Red: LWS cone, green: MWS cone, blue: SWS cone, magenta: UVS cone, black: double cone.

Supplementary Note 3

Spectrophotometer test

In order to corroborate the general finding using UV/VIS photography – that broad-tailed hummingbird (*Selasphorus platycercus*) gorgets change in reflected color with viewing geometry, even under diffuse lighting – further testing was undertaken using a goniometer and spectrophotometer. The goniometer allowed us to take reflectance readings at systematically-varied probe and lighting positions. Readings were taken of a point on the gorget of one male broad-tailed specimen held in the Princeton University bird collection using an OceanOptics USB4000 spectrometer and PX-2 Pulsed Xenon Lamp. Two separate Avantes FC-UV200-2-1.5x100 optic fibers were used for reflectance readings and lighting, along with an Avantes RPH-1 Reflection Probe Holder (goniometer). In a coordinate system identical to that of the main paper (*i.e.*, 0° being normal to the long axis of the bird), reflectance readings were taken at systematically varied angles, from -75° to 30° in steps of 15°. At each reading angle, reflectance was captured at multiple lighting angles, varied systematically from -75° to 75° in steps of 15°. These multiple readings allowed us to simulate reflectance measurements under diffuse lighting at each reading positon (viewing angle) while using the point light source of the spectrometer. This was accomplished for each reading position by dividing the sum of the spectral responses across all lighting positions (subtracting from each a dark current reading) by the sum of spectral responses to a 100% reflectance standard (as measured at the corresponding angles, and subtracting a dark current reading). These calculated reflectance spectra were then submitted to a computational model of hummingbird vision using identical cone sensitivities as the model used in the main paper, using the R package Pavo [9]. The results give a good qualitative fit to the findings in the main paper.

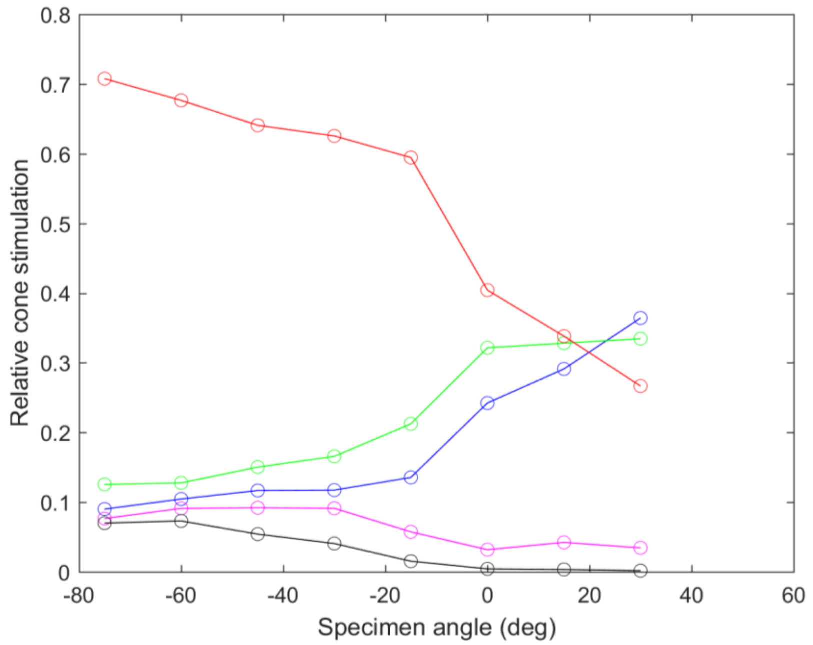

Supplementary Figure 9 – Relative cone stimulations from a point on a male broad-tailed hummingbird gorget measured using a spectrophotometer, simulating readings under diffuse

lighting. The perceived color of the gorget changes with the angle of the viewer in a similar fashion to that estimated using UV/VIS photography in the main paper. Red: LWS cone, green: MWS cone, blue: SWS cone, magenta: UVS cone, black: double cone.

Supplementary Table 3. Museum specimen details.

Common Name	Species Name	Sex	Museum	Number	Alt. Number	Location	Country	Specimen date
broad-tailed hummingbird	*Selasphorus platycercus*	M	AMNH	754575	1274	Arizona	USA	6/27/1917
broad-tailed hummingbird	*Selasphorus platycercus*	M	AMNH	754602	10274	Arizona	USA	6/27/1917
broad-tailed hummingbird	*Selasphorus platycercus*	M	AMNH	754600	10272	Arizona	USA	6/27/1917
broad-tailed hummingbird	*Selasphorus platycercus*	M	AMNH	754593	10265	Arizona	USA	6/13/1917
broad-tailed hummingbird	*Selasphorus platycercus*	M	AMNH	706335	-	Chihuahua	Mexico	6/11/1917
broad-tailed hummingbird	*Selasphorus platycercus*	M	AMNH	361662	-	Arizona	USA	5/11/1917
broad-tailed hummingbird	*Selasphorus platycercus*	M	AMNH	754572	1270	Arizona	USA	6/21/1917
broad-tailed hummingbird	*Selasphorus platycercus*	M	AMNH	754621	11308	Arizona	USA	9/4/1920
broad-tailed hummingbird	*Selasphorus platycercus*	M	AMNH	754640	14048	Chihuahua	Mexico	6/1/1921
broad-tailed hummingbird	*Selasphorus platycercus*	M	AMNH	754590	10261	Arizona	USA	6/12/1919
European robin	*Erithacus rubecula*	M	AMNH	579269	-	Madeira	Portugal	12/1/1910
black-winged red bishop	*Euplectes hordeaceus*	M	AMNH	831117	-	Soroti	Uganda	6/2/1946

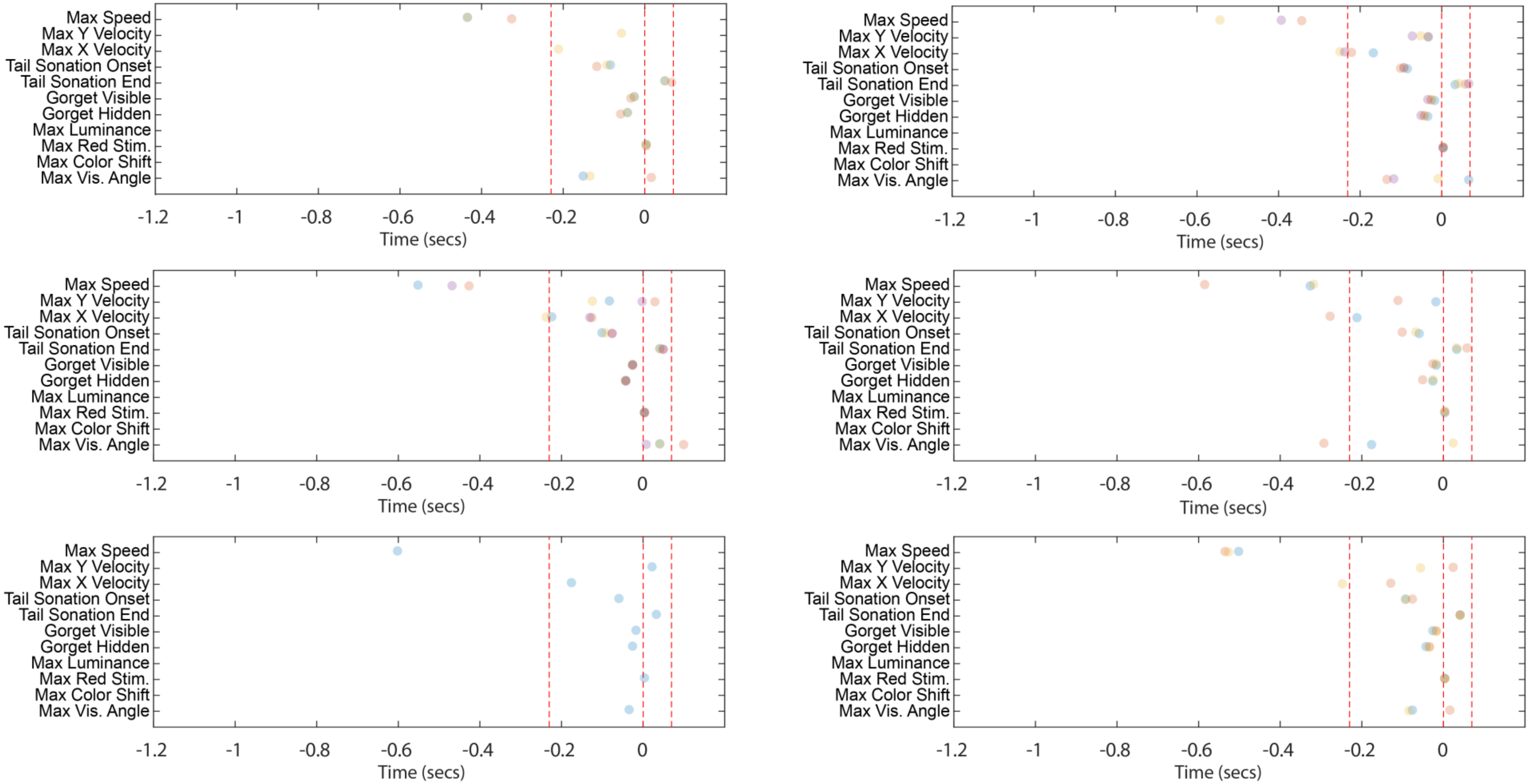
Max Speed
Max Y Velocity
Max X Velocity
Tail Sonation Onset
Tail Sonation End
Gorget Visible
Gorget Hidden
Max Luminance
Max Red Stim.
Max Color Shift
Max Vis. Angle
-1.2
-1
-0.8
-0.6
-0.4
-0.2
0
Time (secs)

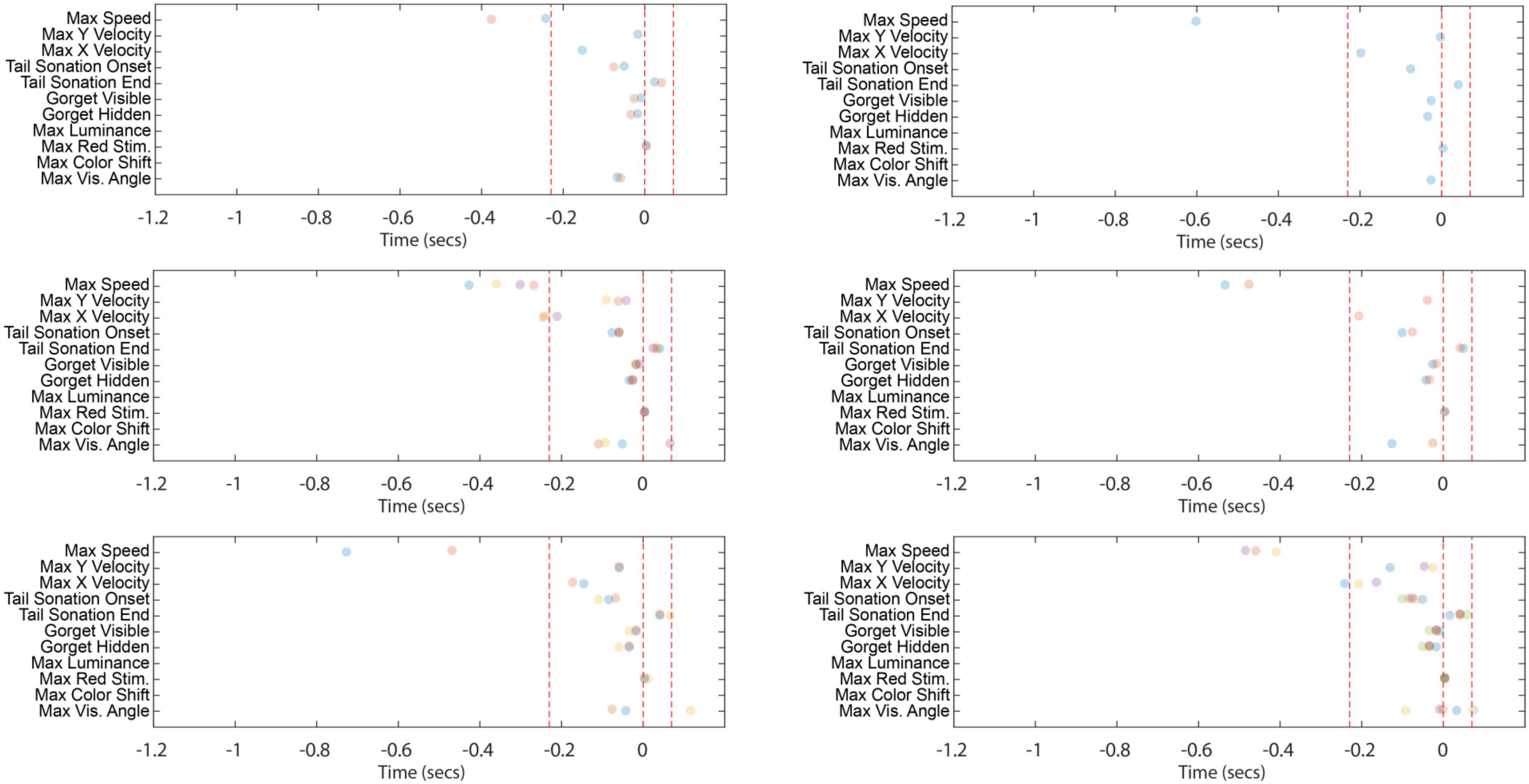
Max Speed
Max Y Velocity
Max X Velocity
Tail Sonation Onset
Tail Sonation End
Gorget Visible
Gorget Hidden
Max Luminance
Max Red Stim.
Max Color Shift
Max Vis. Angle
-1.2
-1
-0.8
-0.6
-0.4
-0.2
0
Time (secs)

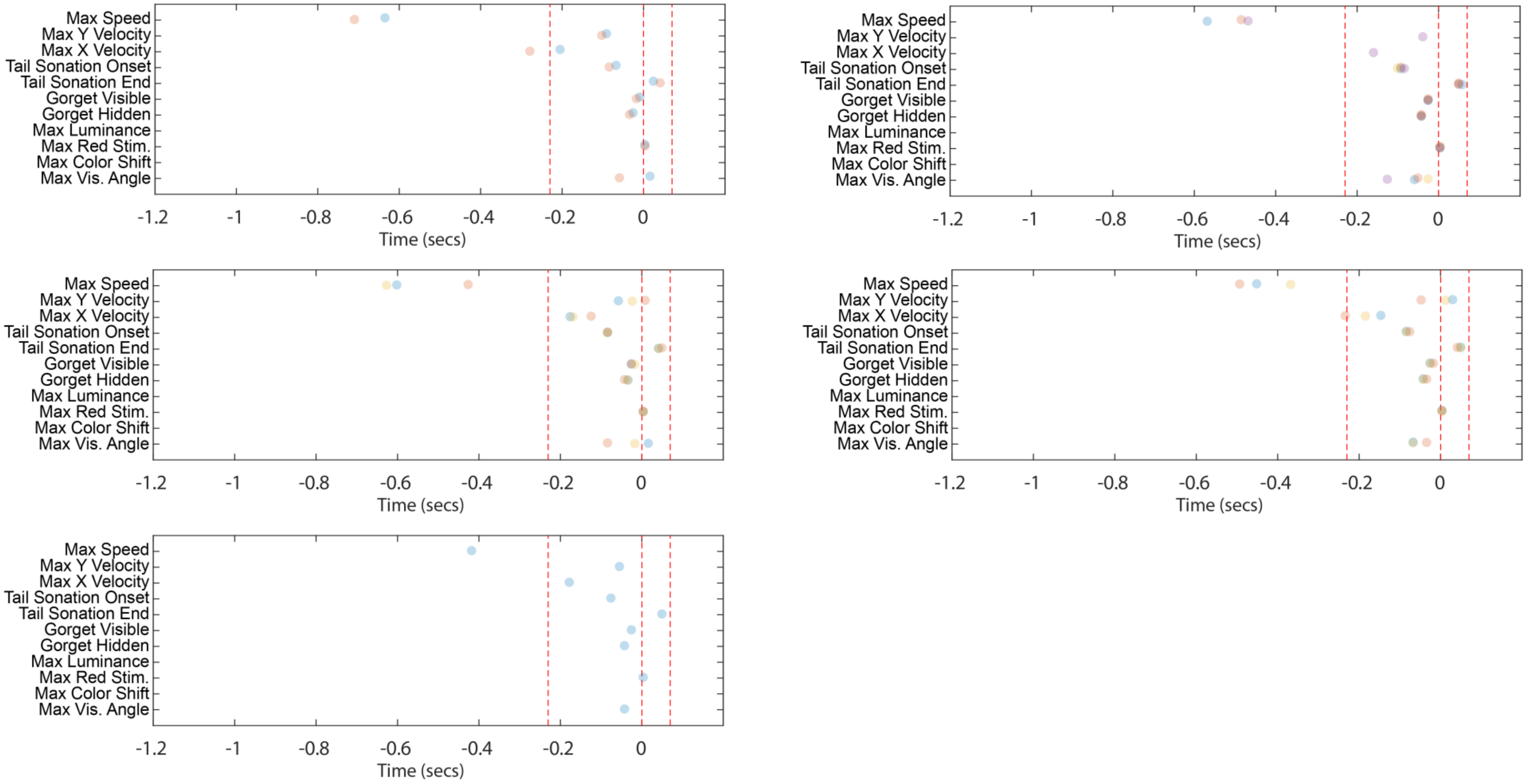

Supplementary Figure 10. Timing of key features of the hummingbird dives per bout – see main paper: time of maximal estimated speed, time of maximal Y (vertical) and X (horizontal) velocity, times of tail-generated sonation onset and cessation, times of gorget visibility, time of maximal estimated luminance of the gorget, time of maximal estimated female LWS stimulation, time of maximal color shift, and the time of maximal female visual angle taken up by the male. Each panel represents one recording which corresponds to one diving bout. Timing of measures for individual dives are indicated with color.

Supplementary References

1. Hedrick, T. L. Software techniques for two- and three-dimensional kinematic measurements of biological and biomimetic systems. *Bioinspir. Biomim.* **3**, 034001 (2008).
2. Schuchmann, K. . & Boesman, P. Broad-tailed Hummingbird (Selasphorus platycercus). in *Handbook of the Birds of the World Alive.* (Lynx Edicions, 2018).
3. Clark, C. J., McGuire, J. A., Bonaccorso, E., Berv, J. S. & Prum, R. O. Complex coevolution of wing, tail, and vocal sounds of courting male bee hummingbirds. *Evolution* **72**, 630–646 (2018).
4. Clark, C. J. & Mistick, E. A. Strategic acoustic control of a hummingbird courtship dive. *Curr. Biol. CB* **28**, 1257-1264.e6 (2018).
5. Clark, C. J. & Feo, T. J. The Anna's hummingbird chirps with its tail: a new mechanism of sonation in birds. *Proc. R. Soc. Lond. B Biol. Sci.* **275**, 955–962 (2008).
6. Stoddard, M. C. & Prum, R. O. Evolution of avian plumage color in a tetrahedral color space: a phylogenetic analysis of new world buntings. *Am. Nat.* **171**, 755–776 (2008).
7. Håstad, O., Victorsson, J. & Ödeen, A. Differences in color vision make passerines less conspicuous in the eyes of their predators. *Proc. Natl. Acad. Sci.* **102**, 6391–6394 (2005).
8. Hart, N. S. Variations in cone photoreceptor abundance and the visual ecology of birds. *J. Comp. Physiol. A* **187**, 685–697 (2001).
9. Maia, R. *et al.* pavo: an R package for the analysis, visualization and organization of spectral data. *Methods Ecol. Evol.* **4**, 906–913 (2013).
